# Molecular Design of Near-IR Dyes with Different Surface Energy for Selective Loading to the Heterojunction in Blend Films

**DOI:** 10.1038/srep09321

**Published:** 2015-03-20

**Authors:** Huajun Xu, Takaaki Wada, Hideo Ohkita, Hiroaki Benten, Shinzaburo Ito

**Affiliations:** 1 Department of Polymer Chemistry, Graduate School of Engineering, Kyoto University, Katsura, Nishikyo, Kyoto 615-8510, Japan; 2 Japan Science and Technology Agency (JST), PRESTO, 4-1-8 Honcho Kawaguchi, Saitama 332-0012, Japan

## Abstract

We have synthesized three silicon phthalocyanine dyes with different hydrophobic substituents in order to control surface energy in the solid state, aiming at selective loading of the dyes into blend films of poly(3-hexylthiophene) (P3HT) and polystyrene (PS). These three dyes are differently located at P3HT domains, at P3HT/PS interface, and at PS domains, respectively, which are fully consistent with the locations predicted by the wetting coefficient derived from the surface energy of each material.

Self-assembling materials play a central role in various functional materials where functional molecules are precisely arranged on a nanometer scale in a required configuration. For this purpose, various approaches such as the self-assembled monolayer method^1^,^2^, Langmuir–Blodgett technique^3^,^4^, layer-by-layer assembly^5^,^6^, and microphase separated assembly^7^,^8^ have been widely applied. In dye-sensitized solar cells, for example, dye molecules are self-assembled to the surface of TiO_2_ nanoparticules, which are covered with iodide/triiodide redox electrolyte solution^9^,^10^. This interfacial location of dye molecules is of particular importance for efficient charge generation because both electron and hole should be transferred from dye to TiO_2_ and the redox couple, respectively. Owing to the elegant molecular alignment, dye-sensitized solar cells based on dye-modified TiO_2_ exhibit highly efficient photovoltaic performance.

Similarly, dye sensitization of polymer solar cells can easily extend the light-harvesting wavelength range^11^,^12^,^13^,^14^,^15^,^16^,^17^,^18^,^19^,^20^,^21^,^22^,^23^,^24^. This approach is simple and versatile and therefore can be easily applied to multi-colored sensitization by incorporating different dye molecules at the same time that have complementary absorption bands in the near-IR region^13^. As is the case with dye-sensitized solar cells, the key to success in dye sensitization of polymer solar cells is selective dye loading to the heterojunction of donor/acceptor interface of blend films because both of hole and electron in dye excitons should be transferred to donor and acceptor materials, respectively, at the same time to generate photocurrent efficiently. Previously, we found that almost all the dye molecules are spontaneously located at the polymer/fullerene interface by analyzing transient absorption dynamics of ternary blend films^14^. Furthermore, we have shown that such spontaneous dye segregation into the interface is partly due to crystallization of polymer, which would expel dye molecules to disordered interface, and partly due to intermediate surface energy of dye molecules, which can minimize interfacial energy when dye molecules are located at the heterojunction^15^. Inspired by these findings, we have motivated to design new dye molecules with appropriate surface energy so that they are spontaneously segregated at specified domains in blend films.

In this study, we synthesized three silicon phthalocyanine derivatives with different axial ligands in order to study the relationship between the surface energy of dye molecules and the loading location in polymer blend films. Here, we selected silicon phthalocyanine bis(tri-*n*-hexylsilyl oxide) (SiPc6) as a standard dye molecule because it serves as an efficient dye sensitizer in polymer/fullerene solar cells as reported previously^12^,^13^,^14^,^15^,^19^. To reduce the surface energy, we synthesized silicon 2,9,16,23-tetra-*tert*-butyl-29*H*,31*H*-phthalocyanine bis(tri-*n*-hexylsilyl oxide) (BuSiPc6), which has four *tert*-butyl groups attached to the phthalocyanine core and two tri-*n*-hexylsilyl oxide groups in the axial ligand. To increase the surface energy, we synthesized silicon phthalocyanine bis(tribenzylsilyl oxide) (SiPcBz), which has two tribenzylsilyl oxide groups in the axial ligand. On the other hand, we employed two amorphous polymers to avoid the dye segregation induced by crystallization of polymer matrix as mentioned above. One polymer is regiorandom poly(3-hexylthiophene) (RRa-P3HT) with a small surface energy^25^,^26^,^27^. The other polymer is polystyrene (PS) with a large surface energy^15^,^27^, which is comparable to that of phenyl-C_61_-butyric acid methyl ester (PCBM) films^15^,^28^. In order to address the location of dye molecules, we measured AFM images of these blend films before and after the films were immersed in pentane solution, which can extract dye molecules selectively.

## Results

### Surface energy

First, we measured contact angles of ultrapure water dropped on several neat films at room temperature in order to evaluate the surface energy of each material by using the Neumann's equation^29^. As summarized in Table 1, the surface energy of matrix materials is evaluated to be 21 mJ m^−2^ (RRa-P3HT) and 26 mJ m^−2^ (PS), which are consistent with previous reports^15^,^25^,^26^,^27^. On the other hand, the surface energy of SiPc derivatives is evaluated to be 20.5 mJ m^−2^ (BuSiPc6), 23 mJ m^−2^ (SiPc6), and 27 mJ m^−2^ (SiPcBz). In other words, the relationship of the surface energy is as follows: BuSiPc6 < SiPc6 < SiPcBz. The lowest surface energy of BuSiPc6 is because the phthalocyanine core ring is surrounded by many hydrophobic alkyl chains: four peripheral *tert*-butyl groups and two tri-*n*-hexylsilyl oxide axial ligands. The highest surface energy of SiPcBz is due to two π-electron rich axial ligands of tribenzylsilyl oxide. As a result, the surface energy of BuSiPc6 is close to that of RRa-P3HT, the surface energy of SiPc6 is in between RRa-P3HT and PS, and the surface energy of SiPcBz is close to that of PS.

### Surface segregation

**Figure 1:**
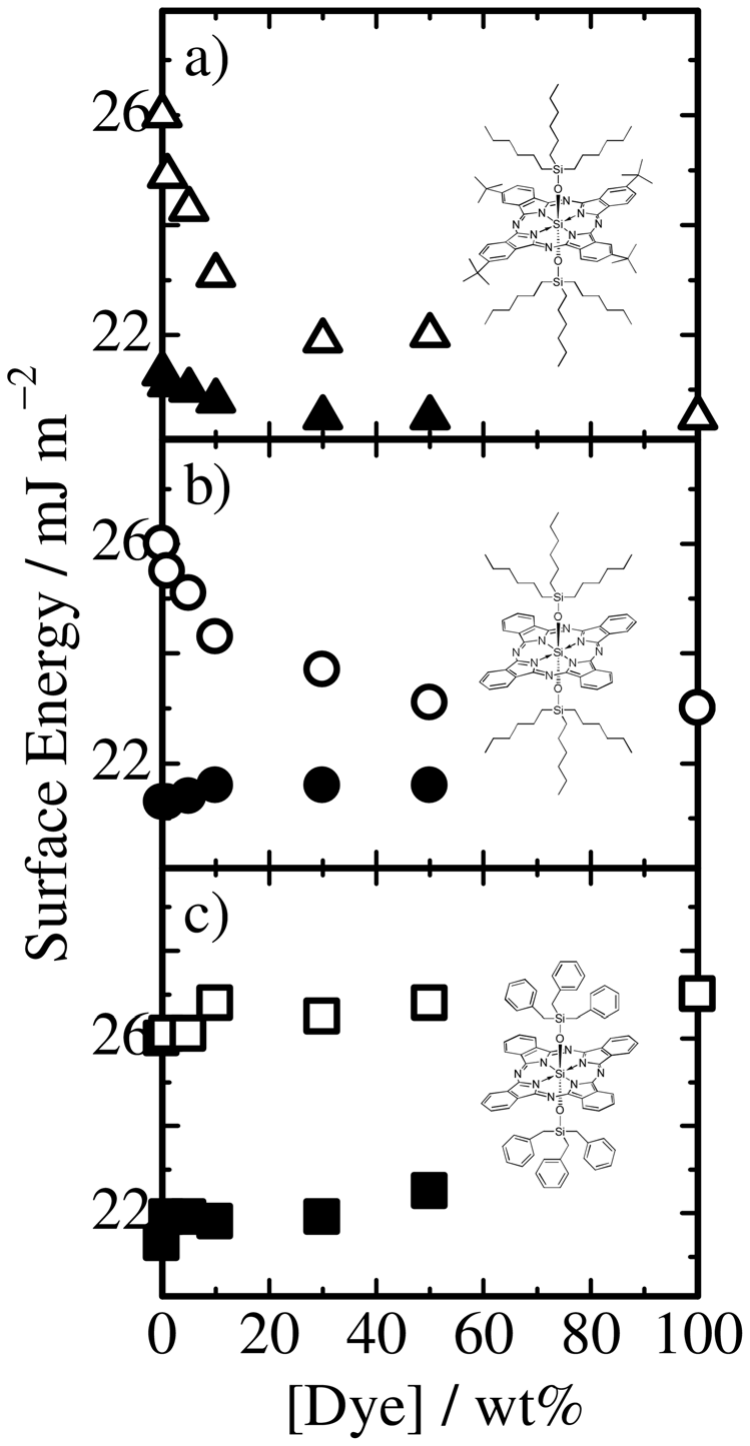
Dependence of surface energy of RRa-P3HT/dye (closed symbols) and PS/dye (open symbols) binary blend films on the dye concentration: a) dye = BuSiPc6, b) dye = SiPc6, and c) dye = SiPcBz.

Next, we measured the dependence of the surface energy of polymer/dye binary blend films on the dye concentration in order to examine how the surface energy of each material impacts the surface segregation in blend films fabricated by spincoating. For BuSiPc6 doped in RRa-P3HT or PS films, as shown in Figure 1a, the surface energy of both blend films not linearly but rapidly decreased from that of polymer neat films and approached that of BuSiPc6 with increasing dye fraction. This is because BuSiPc6 has the lowest surface energy among them and hence can minimize the interface energy at the air/film interface. For SiPc6 doped in RRa-P3HT or PS films, as shown in Figure 1b, the surface energy of RRa-P3HT/SiPc6 remained the same up to 50 wt% dye fraction while that of PS/SiPc6 rapidly decreased to that of SiPc6 with increasing dye fraction. This is because SiPc6 has surface energy in between that of RRa-P3HT and PS. For SiPcBz doped in RRa-P3HT or PS films, as shown in Figure 1c, the surface energy of both blend films remained the same up to 50 wt% dye fraction and finally increased to that of SiPcBz with increasing dye fraction. This is because SiPcBz has the highest surface energy among them and hence is likely to avoid the air surface. In summary, all these results show that the film surface is likely to be spontaneously covered by a material with the lowest surface energy to reduce the interfacial energy at the air/film interface. In other words, the surface energy has critical impact on the surface segregation in binary blend films^30^,^31^,^32^.

### Dye location in blends

We now move onto the segregation of dye molecules in RRa-P3HT/PS/dye ternary blend films. In order to directly observe the location of dye molecules, we measured AFM images of RRa-P3HT/PS/dye ternary blend films before and after immersing the film in pentane solution, which selectively dissolve dye molecules.

**Figure 2:**
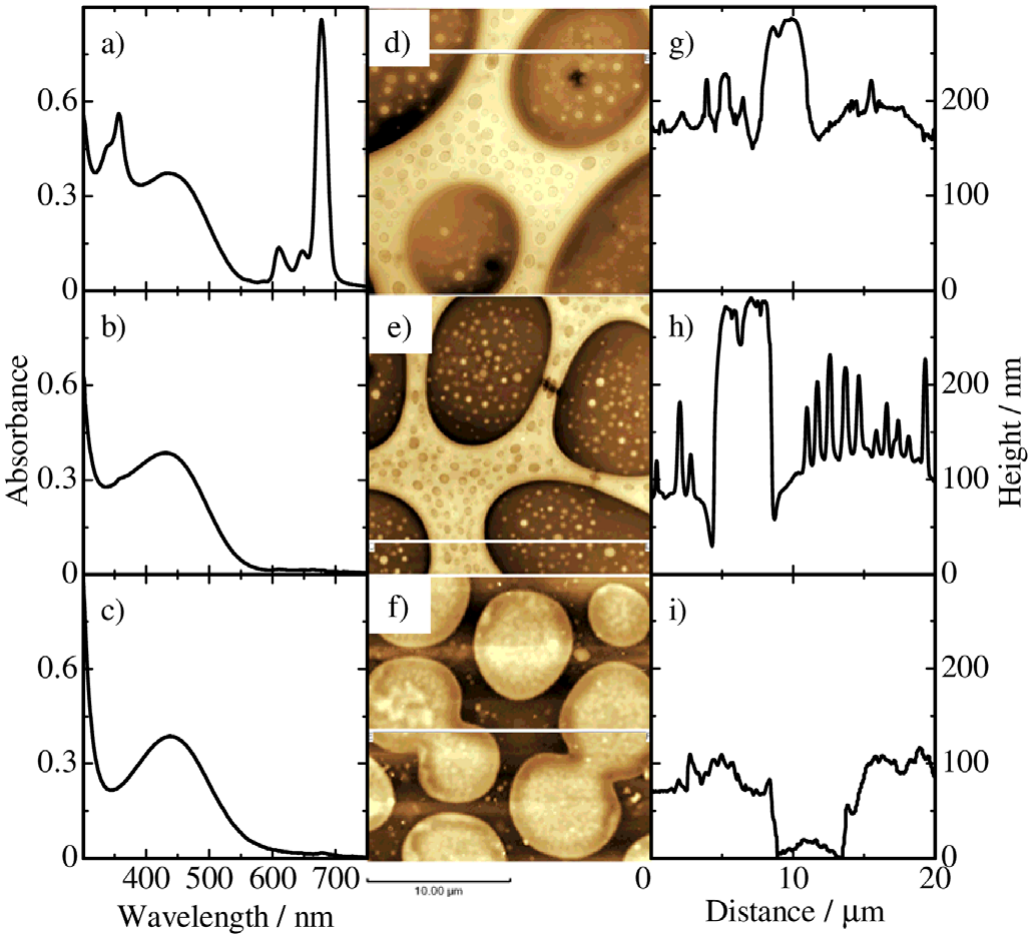
Absorption spectra of RRa-P3HT/PS/BuSiPc6 ternary blend films with a weight ratio of 2:2:1 a) before and b) after the pentane treatment, and c) after the cyclohexane treatment. AFM images of the ternary blend films d) before and e) after the pentane treatment, and f) after the cyclohexane treatment. The cross-sectional line profiles along the white lines indicated in the corresponding AFM images g) before and h) after the pentane treatment, and i) after the cyclohexane treatment.

As shown in Figures 2d and 2e, RRa-P3HT/PS/BuSiPc6 ternary blend films exhibit a similar sea-island structure before and after the pentane treatment. In contrast, there is a clear difference in the cross-sectional line profile before and after the treatment. Here, the cross-section profile is a most typical one in which the height of each domain is roughly evaluated as a difference between the average heights of various sea or island domains and the substrate height. As shown in Figures 2g and 2h, the average height of the island domain is reduced from ~170 to ~120 nm while the height of the sea domains remains the same after the pentane treatment. Consequently, minor components in the island domains are exposed like a pinholder. A slight dip is observed at the sea/island interface after the treatment. As shown in Figures 2a and 2b, dye absorption bands at around 350 and 680 nm completely disappear after the pentane treatment, indicating that all the dye molecules are selectively extracted into pentane solution. After the film is immersed in cyclohexane for 3 min, which dissolves PS selectively as reported previously^15^,^27^,^31^, the sea domains completely disappear while the island domains still remain as shown in Figures 2f and 2i. We therefore conclude that the majority of BuSiPc6 molecules are located in the RRa-P3HT island domains in RRa-P3HT/PS/BuSiPc6 ternary blend films.

**Figure 3:**
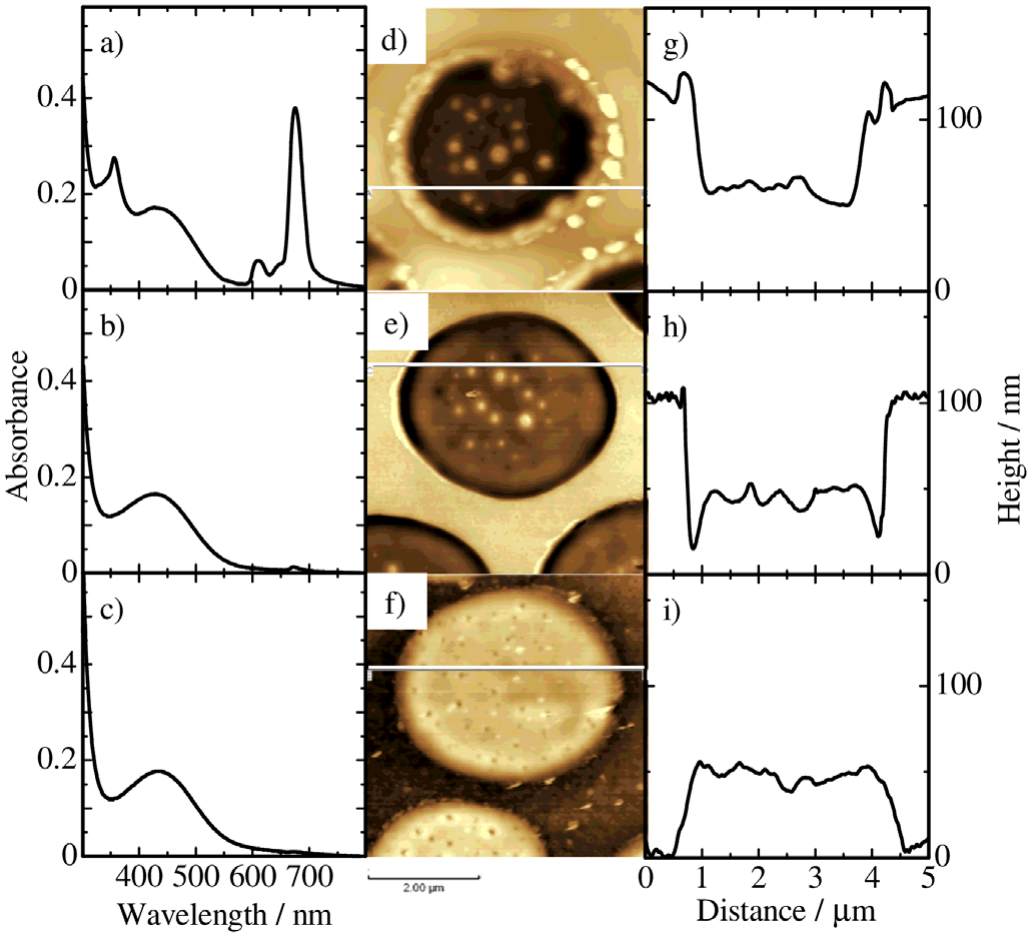
Absorption spectra of RRa-P3HT/PS/SiPc6 ternary blend films with a weight ratio of 2:2:1 a) before and b) after the pentane treatment, and c) after the cyclohexane treatment. AFM images of the ternary blend films d) before and e) after the pentane treatment, and f) after the cyclohexane treatment. The cross-sectional line profiles along the white lines indicated in the corresponding AFM images g) before and h) after the pentane treatment, and i) after the cyclohexane treatment.

As shown in Figures 3d and 3e, RRa-P3HT/PS/SiPc6 ternary blend films also exhibit a similar sea-island structure before and after the pentane treatment. Interestingly, a ring structure is additionally observed at the sea/island interface. In the cross-sectional line profile, as shown in Figures 3g and 3h, the height of the ring portion is higher than that of the other domains before the treatment but sharply depressed after the treatment. No distinct change is found for the other sea/island domains before and after the treatment. As shown in Figures 3a and 3b, dye absorption bands at around 350 and 680 nm completely disappear after the pentane treatment, again indicating that all the dye molecules are selectively extracted into pentane solution. We therefore conclude that the majority of SiPc6 molecules are located at the RRa-P3HT/PS interface in RRa-P3HT/PS/SiPc6 ternary blend films.

**Figure 4:**
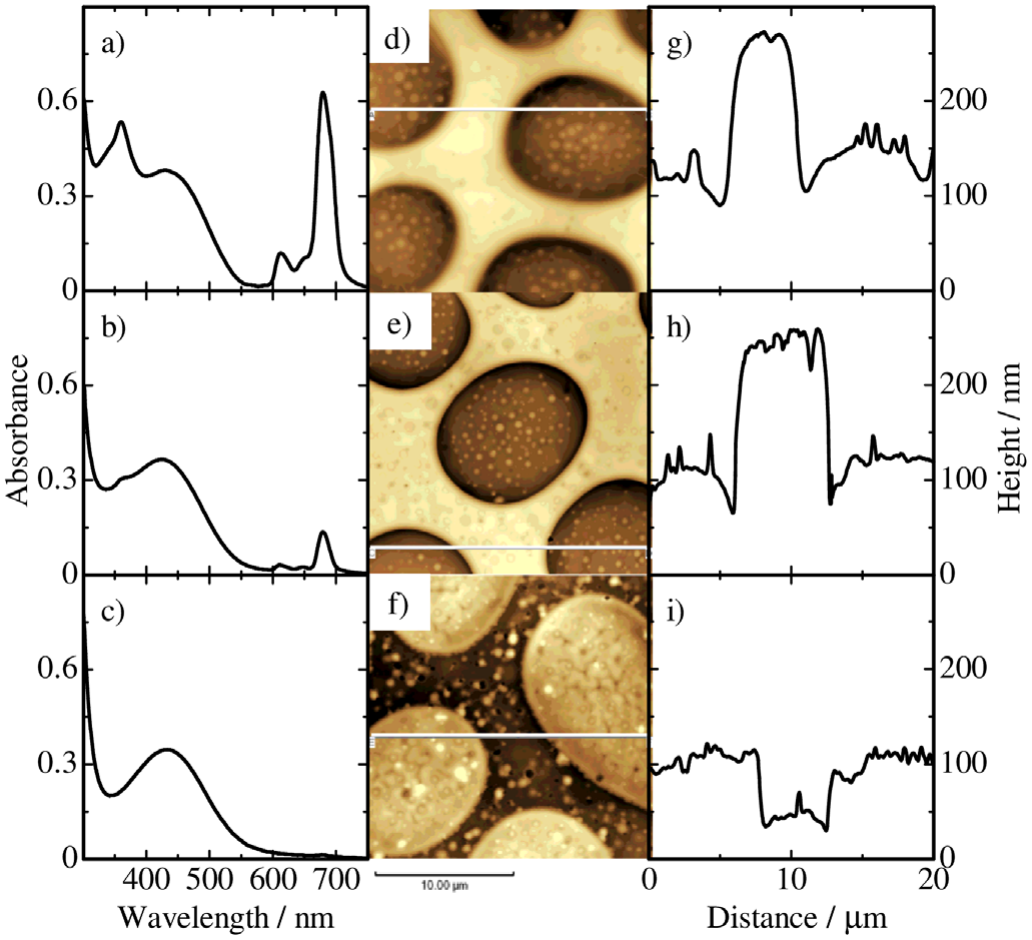
Absorption spectra of RRa-P3HT/PS/SiPcBz ternary blend films with a weight ratio of 2:2:1 a) before and b) after the pentane treatment, and c) after the cyclohexane treatment. AFM images of the ternary blend films d) before and e) after the pentane treatment, and f) after the cyclohexane treatment. The cross-sectional line profiles along the white lines indicated in the corresponding AFM images g) before and h) after the pentane treatment, and i) after the cyclohexane treatment.

As shown in Figures 4d and 4e, RRa-P3HT/PS/SiPcBz ternary blend films also exhibit a similar sea-island structure before and after the pentane treatment. In the cross-sectional line profile, as shown in Figures 4g and 4h, the height of the sea domains is higher than that of the island domains before and after the treatment but slightly reduced from ~270 to ~250 nm after the treatment while the height of the island domains remains the same before and after the treatment. In addition, several dips and a slight dip are observed in the sea domains and at the sea/island interface, respectively, after the treatment. As shown in Figures 4a and 4b, dye absorption bands at around 350 and 680 nm decreases by 75% but still observed even after the pentane treatment, indicating that 75% of dyes are extracted into pentane solution but 25% of dyes still remains in the blend film. After the cyclohexane treatment, as shown in Figures 4c and 4i, the dye absorption bands completely disappear and no height change is observed for the RRa-P3HT domains. These findings indicate that the remaining dye molecules (25%) are removed from the PS domains. We therefore conclude that 75% of SiPcBz molecules are located at the surface of the PS sea domains and the other 25% of SiPcBz are located inside the PS sea domains or the interface in RRa-P3HT/PS/SiPcBz ternary blend films.

## Discussion

For quantitative discussion, we roughly estimate the dye fraction at each location in ternary blend films from the difference in the AFM images and the absorption spectra before and after the pentane treatment. Details of the estimation are described in the Supplementary Information. In RRa-P3HT/PS/BuSiPc6 ternary blend films, as summarized in Table 2, 87% of BuSiPc6 are located at RRa-P3HT domains and the rest of them are located at RRa-P3HT/PS interface. In RRa-P3HT/PS/SiPc6 ternary blend films, 90% of SiPc6 are located at RRa-P3HT/PS interface and the rest of them are located at RRa-P3HT domains. In RRa-P3HT/PS/SiPcBz ternary blend films, 93% of SiPcBz are located at PS domains and the rest of them are located at RRa-P3HT/PS interface. Although these are rough estimation, it can be safely said that the majority of dye molecules are spontaneously located at RRa-P3HT domains in RRa-P3HT/PS/BuSiPc6, at RRa-P3HT/PS interface in RRa-P3HT/PS/SiPc6, at PS domains in RRa-P3HT/PS/SiPcBz ternary blend films. These dye allocations can be predicted in terms of the surface energy of each material as discussed below.

As reported previously, the interfacial energy is one of the key parameters for such spontaneous segregation of the third material in ternary blend films^15^,^33^,^34^,^35^,^36^. Sumita et al. introduced a key parameter of the wetting coefficient (*ω*) evaluated from the surface energy of each component material to predict allocation of fillers doped as the third material in polymer/polymer binary blends. Here, the surface energy of a matrix polymer A is lower than that of the other matrix polymer B (*γ*_A_ < *γ*_B_). The third material filler will be located at polymer A domains when *ω* > 1, at the A/B interface when −1 < *ω* < 1, and at polymer B domains when *ω* < −1. On the basis of this simple model, we calculate the wetting coefficient of dye (*ω*_dye_) to predict the dye location in RRa-P3HT/PS/dye ternary blend films. As a result, dye molecules are predicated to be located at RRa-P3HT domains (BuSiPc6, *ω*_dye_ = 1.2), at the RRa-P3HT/PS interface (SiPc6, *ω*_dye_ = 0.2), and at PS domains (SiPcBz, *ω*_dye_ = −1.4). These locations predicted are, as shown in Table 2, fully consistent with the dye location revealed by the AFM measurements.

In conclusion, we demonstrated that dye molecules can be selectively loaded into each domain or interface in polymer/polymer blend films by careful designing the surface energy of dye with appropriate axial ligands. We thus emphasize that our finding can provide a molecular design perspective for selective loading of small molecules in blend films fabricated even by solution processes such as spin-coating. In particular, this strategy is useful for developing new dye molecules employed in ternary blend polymer solar cells, in which interfacial dye loading is the key to success for efficient dye sensitization.

## Methods

### Dye synthesis

The reaction schemes and the chemical structures of SiPc derivatives with various axial groups employed in this study are summarized in the Supporting Information.

BuSiPc6: A mixture of silicon 2,9,16,23-tetra-*tert*-butyl-29*H*,31*H*-phthalocyanine dihydroxide (BuSiPc(OH)_2_) (115 mg), chlorotri-*n*-hexylsilane (250 μL), and dry pyridine (15 mL) was refluxed for 6 h. After the solution obtained had been allowed to cool, the solvent was evaporated and chloroform was added to the residue. The solution was washed with saturated NaCl solution, and then dried over MgSO_4_. After evaporation of the solvent, the residue was purified by silica gel column chromatography (toluene/hexane = 1/1 (v/v) as eluent) to afford BuSiPc6 (76.8 mg) as a bluish-green solid (yield = 38%). UV–visible (toluene): *λ*_max_ 669 nm (ε = 2.6 × 10^5^ M^−1^ cm^−1^); ^1^H NMR (400 MHz, CDC1_3_):δ = 9.61–9.64 (m, α-Pc, 8H), 8.35–8.37 (m, β-Pc, 4H), 1.82–1.83 (m, tBu, 36H), 0.67–0.71 (m, ε-CH_2_, 12H), 0.47–0.51 (t, CH_3_, 18H), 0.35–0.42 (m, δ-CH_2_, 12H), 0.03–0.04 (m, γ-CH_2_, 12H), −1.23– −1.29 (m, β-CH_2_, 12H), −2.40– −2.45 (m, α-CH_2_, 12H). MALDI-TOF: *m/z* 1363.9 (M + H). Calcd for C_84_H_126_N_8_O_2_Si_3_: *m/z* 1364.2.

SiPc6: A mixture of silicon phthalocyanine dihydroxide (SiPc(OH)_2_) (85 mg), chlorotrihexylsilane (200 μL), and dry pyridine (10 mL) was refluxed for 6 h. After the solution obtained had been allowed to cool, the solvent was evaporated and chloroform was added to the residue. The solution was washed with saturated NaCl solution, and then dried over MgSO_4_. After evaporation of the solvent, the residue was purified by silica gel column chromatography (toluene/hexane = 1/1 (v/v) as eluent) to afford SiPc6 (106 mg) as a blue solid (yield = 62.8%). UV–visible (toluene): *λ*_max_ 668 nm (ε = 3.0 × 10^5^ M^−1^ cm^−1^); ^1^H NMR (400 MHz, CDC1_3_):δ = 9.63 (m, 3,6-Pc, 8H), 8.31 (m, 4,5-Pc, 8H), 0.81 (m, ε-CH_2_, 12H), 0.71 (t, CH_3_, 18H), 0.36 (m, δ-CH_2_, 12H), 0.02 (m, γ-CH_2_, 12H), −1.28 (m, β-CH_2_, 12H), −2.45 (m, α-CH_2_, 12H). MALDI-TOF: *m/z* 1139.7 (M + H). Calcd for C_68_H_94_N_8_O_2_Si_3_: *m/z* 1139.8.

SiPcBz: A mixture of silicon phthalocyanine dihydroxide (SiPc(OH)_2_) (99 mg), chlorotribenzylsilane (270 mg), and dry pyridine (15 mL) was refluxed for 5 h. After the solution obtained had been allowed to cool, the solvent was evaporated and chloroform was added to the residue. The solution was washed with saturated NaCl solution, and then dried over MgSO_4_. After evaporation of the solvent, the residue was purified by silica gel column chromatography (dichloromethane/acetone = 30/1 (v/v) as eluent) to afford SiPcBz (126 mg) as a bluish-green solid (yield = 62%). UV−visible (toluene): *λ*_max_ 675 nm (ε = 3.0 × 10^5^ M^−1^ cm^−1^); ^1^H NMR (400 MHz, CDC1_3_):δ = 9.58–9.63 (m, 3,6-Pc, 8H), 8.21–8.37 (m, 4,5-Pc, 8H), 6.45–6.53 (m, 4-Ph, 6H), 6.28–6.39 (m, 3,5-Ph, 12H), 4.81–4.98 (m, 2,6-Ph, 12H), −0.96 (m, CH_2_, 12H). MALDI-TOF: *m/z* 1175.4 (M + H). Calcd for C_74_H_58_N_8_O_2_Si_3_: *m/z* 1175.6.

### Sample fabrications

The quartz glass or glass substrates were cleaned by ultrasonication in toluene, acetone, and ethanol each for 15 min, dried with N_2_, and cleaned with a UV–O_3_ cleaner for 30 min. For contact angle measurements, neat films (~100 nm) of RRa-P3HT (Aldrich, head-to-head:head-to-tail = 1:1, *M*_w_ = 90600), PS (Scientific Polymer Products, *M*_w_ = 22000), and SiPc dyes were individually spin-coated on the cleaned quartz glass. For the dye-concentration dependence measurement, RRa-P3HT and PS films doped with dyes at various concentrations were prepared by spin-coating on the glass substrate from a chlorobenzene solution to give blend films with a thickness of about 80 nm. For the AFM measurements, RRa-P3HT/PS/dye ternary blend films were spin-coated from a chlorobenzene solution at a weight ratio of 2:2:1 (10:10:5 mg mL^−1^).

### Measurements

For materials characterization, ^1^H NMR spectra were recorded on a JEOL EX-400 spectrometer at 400 MHz by using CDCl_3_ as solvent and MALDI-TOF mass spectra were obtained on a Bruker Ultraflex MALDI/TOF mass spectrometer with dithranol as matrix. To evaluate the surface energy of each material, contact angle *θ*_X_ was measured on the neat film of material X using ultrapure water at room temperature. The surface energy *γ*_X_ can be evaluated from *θ*_X_ by the Neumann's equation combined with the Young's equation^15^,^29^. From the surface energy *γ*_X_ of each material, the interface energy *γ*_AB_ between A and B materials was evaluated by the Neumann's equation^15^,^29^. The wetting coefficient *ω*_C_ of material C in a matrix blend of A and B can be evaluated from the interface energy: *ω*_C_ = (*γ*_BC_ − *γ*_AC_)/*γ*_AB_^29^,^33^. Therefore, the dye molecule location can be predicted from the wetting coefficient *ω*_C_ in a blend film. Absorption spectra of the blend films were measured with a spectrophotometer (Hitachi, UV-3500). Film morphology and thickness of blend films were measured with AFM (Shimadzu, SPM-9600) in the contact mode. The AFM images were observed for as-cast films, SiPc dye-removed films, and PS-removed films. The SiPc dye-removed films were obtained by immersing the as-cast films into pentane solution (Wako) for 1 min. The PS-removed films were obtained by immersing the SiPc dye-removed films into cyclohexane (Nacalai Tesque) for 3 min.

## Author Contributions

H.O. and S.I. conceptualized and directed the research project. H.X. and T.W. synthesized the SiPc dyes and performed measurements. H.X. wrote the original manuscript with the assistance of H.O. All authors discussed the results and commented on the manuscript.

## Supplementary Material

Supplementary Information SupplementaryInformation

## Figures and Tables

**Table 1:** Contact angle and surface energy for various neat films

Materials	Contact angle/deg	Surface energy/mJ m^−2^
BuSiPc6	104	20.5
RRa-P3HT	103	21
SiPc6	100	23
PS	95	26
SiPcBz	93	27
PCBM	91	29

**Table 2:** Dye location fraction in RRa-P3HT/PS blend films

Dye	RRa-P3HT	Interface	PS	Predicted Location
BuSiPc6	87%	13%	—	RRa-P3HT
SiPc6	10%	90%	—	Interface
SiPcBz	—	7%	93%a)	PS

^a)^Surface 68%, Inside 25%
