# Supplementary material for: Molecular Design of Near-IR Dyes with Different Surface Energy for Selective Loading to the Heterojunction in Blend Films
Source: Sci Rep. 2015 Mar 20;5:9321. doi: 10.1038/srep09321 (PMC4366806; doi:10.1038/srep09321)
Supplement: Supplementary Information — SupplementaryInformation [file srep09321-s1.doc]

*Supplementary Information*

**Molecular Design of Near-IR Dyes with Different Surface Energy for Selective Loading to the Heterojunction in Blend Films**

Huajun Xu,^†^ Takaaki Wada,^†^ Hideo Ohkita,^†,‡,*^ Hiroaki Benten,^†^ Shinzaburo Ito^†^

^†^Department of Polymer Chemistry, Graduate School of Engineering,

Kyoto University, Katsura, Nishikyo, Kyoto 615-8510, Japan

^‡^Japan Science and Technology Agency (JST), PRESTO,

4-1-8 Honcho Kawaguchi, Saitama 332-0012, Japan

**Synthesis of Dye Derivatives**

Figure S1 shows the reaction schemes and the chemical structures of SiPc derivatives with various axial groups employed in this study. The axial substituents are summarized in Table S1.

**Figure S1.** Synthetic scheme of SiPc derivatives.

**Table S1.** Substituents of SiPc derivatives.

| Dye | BuSiPc6 | SiPc6 | SiPcBz |
| --- | --- | --- | --- |
| R_1_ | -C_6_H_13_ | -C_6_H_13_ | -CH_2_(C_6_H_5_) |
| R_2_ | -C(CH_3_)_3_ | -H | -H |

**Estimation of Dye Fraction**

The dye fraction in each domain is roughly estimated on the basis of the AFM images before and after the pentane treatment. Table S2 summarizes the average thickness of each domain (sea, interface, and island) before and after the pentane treatment evaluated for at least 18 domains in ternary blend films.

**Table S2.** The average thickness of each domain before and after the pentane treatment.

| RRa-P3HT/PS/Dye  (Average height / nm) | BuSiPc6 | | | SiPc6 | | | SiPcBz | | |
| --- | --- | --- | --- | --- | --- | --- | --- | --- | --- |
|  | Sea | Island | Ring | Sea | Island | Ring | Sea | Island | Ring |
| Before | 270 | 170 | 150 | 115 | 60 | 130 | 270 | 120 | 100 |
| After | 270 | 120 | 50 | 115 | 55 | 30 | 250 | 120 | 70 |

For RRa-P3HT/PS/BuSiPc6 blend films, the average diameter of P3HT circular island domains was ~9 μm and the average width of the interfacial ring dip was ~1 μm. In other words, the average area of one P3HT domain and the surrounding interfacial ring dip is estimated to be ~63.5 μm^2^ and ~4.75 μm^2^, respectively. As shown in Figure 3, the height of the P3HT domains was reduced by ~50 nm from ~170 to ~120 nm, and the height of the interfacial ring dip was reduced by ~100 nm from ~150 to ~50 nm. Thus, the decrease in the volume was estimated to be Δ*V* = ~3.2 μm^3^ for one P3HT circular domain and to be Δ*V* = ~0.5 μm^3^ for the interfacial ring dip. Here, BuSiPc6 molecules located at the PS domains are negligible because no distinct change was observed for the surface of the PS domains before and after the pentane treatment. Therefore, the dye fraction in RRa-P3HT/PS/BuSiPc6 is evaluated to be 87 vol% for one P3HT circular domain 13 vol% for the interfacial ring dip.

For RRa-P3HT/PS/SiPc6 blend films, the average diameter of the P3HT circular island domains was ~3 μm and the average width of the interfacial ring dip was ~0.7 μm. In other words, the average area of one P3HT domain and the surrounding interfacial ring dip is estimated to be ~7.1 μm^2^ and ~3.7 μm^2^, respectively. As shown in Figure 4, the height of the P3HT domains was reduced by ~5 nm from ~60 to ~55 nm, and the height of interfacial ring dip was reduced by ~100 nm from ~130 to ~30 nm. Thus, the decrease in the volume was estimated to be Δ*V* = ~0.04 μm^3^ for one P3HT circular domain and to be Δ*V* = ~0.37 μm^3^ for the interfacial ring dip. Here, SiPc6 molecules located at the PS domains are negligible because no distinct change was observed for the surface of the PS domains before and after the pentane treatment. Therefore, the dye fraction in RRa-P3HT/PS/SiPc6 is evaluated to be 10 vol% for one P3HT circular domain 90 vol% for the interfacial ring dip.

For RRa-P3HT/PS/SiPcBz blend films, the average diameter of the P3HT circular island domains was ~9 μm and the average width of the interfacial ring dip was ~1 μm. In other words, the average area of one P3HT domain and the surrounding interfacial ring dip is estimated to be ~63.5 μm^2^ and ~4.75 μm^2^, respectively. There were typically about three P3HT domains in the AFM images (20 μm × 20 μm). Thus, the remaining area of the PS domains is estimated to be 20 μm × 20 μm – 3 × (63.5 μm^2^ + 4.75 μm^2^) = 195 μm^2^, which is roughly half the total area, and consistent with the weight ratio of RRa-P3HT : PS = 1 : 1. As shown in Figure 5, the height of the PS domains was reduced by ~20 nm from ~270 to ~250 nm, and the height of the interfacial ring dip was reduced by ~30 nm from ~100 to ~70 nm. No distinct change was observed for the surface of the P3HT domains before and after the pentane treatment, suggesting that SiPc6 molecules located at the P3HT domains are negligible. Thus, the decrease in the volume was estimated to be Δ*V* = ~3.9 μm^3^ for the PS sea domain and to be Δ*V* = ~0.43 μm^3^ for the interfacial ring dip. Therefore, the dye fraction in RRa-P3HT/PS/SiPcBz is evaluated to be 68 vol% (75 × 3.9/(3.9 + 0.43)) for the surface of the PS sea domain (extracted fraction) 25 vol% for the inside of the sea domain (remaining fraction), and 7 vol% (75 × 0.43/(3.9 + 0.43)) for the interfacial ring dip.
